# Evidence Limitations in Determining Sexually Dimorphic Outcomes in Pediatric Post-Traumatic Hypopituitarism and the Path Forward

**DOI:** 10.3389/fneur.2020.551923

**Published:** 2020-11-26

**Authors:** Alina Nico West, Alicia M. Diaz-Thomas, Nadeem I. Shafi

**Affiliations:** ^1^Division of Critical Care Medicine, Department of Pediatrics, University of Tennessee Health Science Center, Memphis, TN, United States; ^2^Division of Endocrinology, Department of Pediatrics, University of Tennessee Health Science Center, Memphis, TN, United States

**Keywords:** Pediatric hypopituitarism, neurotrauma, sexual dimorphism, neuroendocrine dysfunction, TBI, neuropsychology, neurocognition, adverse childhood events

## Abstract

Neuroendocrine dysfunction can occur as a consequence of traumatic brain injury (TBI), and disruptions to the hypothalamic-pituitary axis can be especially consequential to children. The purpose of our review is to summarize current literature relevant to studying sex differences in pediatric post-traumatic hypopituitarism (PTHP). Our understanding of incidence, time course, and impact is constrained by studies which are primarily small, are disadvantaged by significant methodological challenges, and have investigated limited temporal windows. Because hormonal changes underpin the basis of growth and development, the timing of injury and PTHP testing with respect to pubertal stage gains particular importance. Reciprocal relationships among neuroendocrine function, TBI, adverse childhood events, and physiological, psychological and cognitive sequelae are underconsidered influencers of sexually dimorphic outcomes. In light of the tremendous heterogeneity in this body of literature, we conclude with the common path upon which we must collectively arrive in order to make progress in understanding PTHP.

## Introduction

The pituitary gland sits within the sella turcica, connected to the hypothalamus by the infundibulum and surrounded by a rich vascular web (1). From here, it contributes to maintaining physiologic homeostasis and regulating processes of growth and development. Despite this privileged location, pituitary function can be disrupted by traumatic brain injury (TBI), which directly impacts the pituitary gland or affects its function indirectly via insult to the hypothalamus. Hemorrhage, infarction, and shearing injury lesions can cause hypoxic insults, induce an inflammatory cascade, and upset neuronal function. These mechanisms can impact the anterior and posterior pituitary lobes, infundibulum, pituitary capsule, pars intermedia, and hypothalamus (2). The pituitary volume in the sella can decrease due to pituitary necrosis and/or increased intracranial pressure (3–5). Specific cell types (gonadotrophs and somatotrophs more often than thyrotrophs, corticotrophs, and the axonal projections of the magnocellular neurosecretory cells) can be differentially affected, depending on location and type of injury (3, 6). The constellation of hormonal deficiencies which can occur due to hypothalamic/pituitary dysfunction are referred to as post-traumatic hypopituitarism (PTHP).

Dysfunction of anterior and/or posterior pituitary hormonal axes can cause diabetes insipidus (DI), secondary adrenal insufficiency, central hypothyroidism, precocious puberty, or hypogonadotropic hypogonadism, and growth hormone deficiency (GHD). Single or multiple hormonal deficiencies can occur with transience or persistence. Overall PTHP prevalence estimates range from 5 to 61% in children (1, 7), reflecting tremendous heterogeneity in injury patterns, study populations, and diagnostic approaches.

We embarked upon this review with the goal of describing sex differences in PTHP but found the confounders to be substantial. One biological challenge is that pituitary function and physiologic response vary developmentally by pubertal stage. The major epidemiological challenge is a significant male predominance in TBI patients, creating imbalanced data sets which preclude meaningful statistical comparisons. The male predominance likely contributes to the paucity of studies which have compared neuroendocrine outcomes by sex, particularly in younger age groups. The injury itself presents a challenge because TBI is a very heterogeneous disease, and patient demographics, injury severities, injury mechanisms and causes, treatment modalities, recovery periods, and measured outcomes vary across studies (8, 9). Finally, there is an assortment of challenges relating to investigational approach which we will discuss.

In our review, we first describe how sex influences physical and cognitive development (section “Sexual Dimorphism in Physical and Cognitive Development”), which is followed by how outcomes after TBI vary according to sex (section “Sex-Related Differences in Overall TBI Outcomes”). Together, these essentially serve as co-variates in the sexual dimorphism of PTHP.

A discussion of the various studies of PTHP disorders comprises the section, “The Current State of Pediatric Neuroendocrinopathies Following TBI”. Specific neuroendocrine disorders in the context of pediatric TBI are the focus of our review; however, details of some key adult studies have been included to complement pediatric results. We provide a relatively limited discussion of PTHP in the acute phase of TBI for several reasons. The acute phase often has many confounders including medications such as etomidate and dopamine. The transience of acute phase hypopituitarism can be quite variable and remains poorly described, making it difficult to systematically assess. We focus on the chronic phase because outside of severe life-threatening hemodynamic instability and electrolyte abnormalities, the clinical impacts of hypopituitarism gain relevance after patient survival is assured.

In the section “Reciprocal Influences Among Common TBI and PTHP-Related Consequences”, we touch upon other medical and non-medical factors which relate to both TBI generally and PTHP specifically, creating the possibility of reciprocally influencing outcomes. Finally, after demonstrating how the lack of prospective, sex-balanced, intentional studies hamper the ability to draw conclusions, we end by proposing a path forward (section “Conclusions and the Path Forward”). This path—if agreed upon and adopted in future investigations—would allow us to gain critical insights into the PTHP disorders.

## Sexual Dimorphism in Physical and Cognitive Development

Stereotyped changes in hormonal programming which occur pre-/perinatally and during puberty can impact physical and cognitive development. Increases in the gonadotropins, luteinizing hormone (LH) and follicle-stimulating hormone (FSH), occur in fetal development and in the months following birth. Fetal testosterone levels increase up to 20 weeks gestation, followed by a hypothalamic-pituitary-gonadal axis quiescence from 20 weeks gestation to delivery (10). As a result, both males and females typically experience a “mini-puberty” at ages 1–6 months (11, 12) defined by a rise in testosterone or estradiol, respectively. Following the postnatal period there is gonadotropic suppression until pubarche, which is the earliest sign of pubertal onset. Gonadarche occurs when gonadotropins are released in an increasingly pulsatile fashion. Growth hormone (GH) and insulin growth factor-1 (IGF-1) levels during puberty usually reflect the changes in gonadotropin secretion, increasing as puberty progresses (13). Females enter puberty earlier than males, with age ranges for Tanner stage 2 breast development being 8–12 years and for testicular enlargement to ≥4 mL being 9–14 years (means 9.5 vs. 11.5 years, respectively) (14, 15). The tempo of puberty itself can also vary among individuals. Thyroid hormone (TH) contributes to overall physiological development (13) but sex differences in this axis have not been observed.

Hormonal secretion during growth aligns with normal cognitive changes in brain development. Cognitive features are associated, in part, with hypothalamic-pituitary-gonadotropin feedback mechanisms during puberty. Sexual dimorphism exists in cognitive tasks during adolescence—females having enhanced memory, language, and physiological reactions to stress, while males having more developed visuospatial processing, emotional coping, and sensorimotor feedback. These are reflected in differing brain volumes designated to the task-specific areas (16). We can speculate that fetal testosterone exposures may also influence outcomes including changes in neuroanatomical structures such as rightward corpus callosal asymmetry associated with empathy, language, and visuospatial processing, and in areas of gray matter linked to empathy, language, and social attention (10, 17, 18).

The hormonal changes described above create landscapes which evolve over time and become disparate between the sexes. The stereotyped progression, however, frames periods of time in which the hormonal milieus may be sufficiently similar to allow comparisons of neuroendocrine outcomes—namely, in the pre-, peri-, and post-pubertal stages. (Peri- and post-menopausal periods are occasionally referenced in this review as distinct, late post-pubertal stages in the female hormonal lifespan.) It is worth noting that TBI may disrupt pubertal progression in unpredictable ways (1), further complicating analyses. Measures of cognitive function are more frequently included as part of quality of life-based TBI outcomes, making patterns of sexual dimorphism increasingly relevant.

## Sex-Related Differences in Overall TBI Outcomes

Past reviews have addressed sex differences in mortality and morbidities after TBI including neurological, endocrine, neuropsychological, psychiatric, and cognitive responses to injury stress [for instance, see (19–22)]. The incidence of pediatric TBI is bimodal, with one peak occurring between infancy and 4 years of age, and a second around age 15 years (23). This means that TBI occurrence is low peri-pubertally and low again following brain development during young adulthood. Pubertal females have a 0.78 (95% CI 0.65–0.93) times lower TBI-associated mortality than males (24) with no difference between post-pubertal males and females (adjusted OR = 1.09, 95% CI 0.99–1.21) (25). Males have an overall mortality rate 3 times higher than females (23). There is conflicting data on whether sex is associated with mortality in the pre-pubertal stage—studies have either found no association or a higher mortality rate in females (24, 26). Pre-pubertal and pubertal females have increased ICU admissions and lengths of stay (LOS) (24); however, post-pubertal, perimenopausal, and postmenopausal women have lower hospital and ICU LOS than their male counterparts (25). Interestingly, adult men and women have different frequencies of post-concussive symptoms such as headache, dizziness, irritability, and insomnia, as well as in functional outcomes measured by the Glasgow Outcome Scales—Extended (GOS-E); however, this data has not yet been reported in children based on pubertal stage (27, 28).

## The Current State of Pediatric Neuroendocrinopathies Following TBI

### Current Data on Post-Traumatic Hypopituitarism

We have included 15 pediatric studies in our review, which represent a total of 765 patients. Table 1 provides an overview of the characteristics of these studies−13 are cohort studies reporting PTHP incidence; 2 are cross-sectional, reporting prevalence. Pediatric neuroendocrinopathies have been evaluated in varying sample sizes (*n* = 14–198 patients), with the largest study being by Heather et al. (30). Inclusion criteria of these studies such as age and pubertal stage were broad. Patient ages ranged from 0.1 to nearly 27 years. Four of the 15 studies enrolled within a narrow range of pubertal stages, with 1 focusing on pre-pubertal, and 3 combining pre-pubertal and pubertal; the remainder of studies were non-selective in this regard. These features alone may mask or dampen any estimation of sexual dimorphisms across pubertal stages. Furthermore, all studies had a significant male predominance, with the % female enrollment ranging from 17 to 42%. Interestingly, only 2 studies used a non-TBI control group to compare neuroendocrine testing (29, 43) and 5 used quality of life or functional questionnaire (34, 36, 38, 41, 43) responses.

**Table 1:** PTHP study characteristics.

**References**	**Age range (years)**	**Pubertal stage**	**Sample size**	**Controls Y/N (n)**	**% Females**
Niederland et al. (29)	11.5 ± 0.8^‡^	Pre- and pubertal	26	Y (21)	35
Heather et al. (30)	8.3 ± 3.3^‡^	Pre- and pubertal	198	N	41
Bellone et al. (31)	0.1–14.2	Pre- and pubertal	70	N	17
Auble et al. (32)	2–9	Pre-pubertal^*^	14	N	21
Einaudi et al. (33)	0.3–15.5	All	34^††^	N	21^††^
Poomthavorn et al. (34)	0.1–20.1	All	33^††^	N	36^††^
Kaulfers et al. (35)	1.5–18	All	31	N	42
Srinivas et al. (36)	1–17	All	37	N	27
Norwood et al. (37)	8–21	All	32	N	38
Khadr et al. (38)	5.4–21.7	All	33	N	24
Casano-Sancho et al. (39)	0.2–19.9	All	37	N	19
Personnier et al. (40)	0.8–15.2	All	87	N	31
Salomón-Estébanez et al. (41)	2.7–15.1	All	36	N	39
Dassa et al. (42)	4.2–21.8	All	66	N	26
Daskas et al. (43)	11.3–26.6	All	31	Y (17)	35

‡ *Age range not available (expressed as mean ± SD)*.

* *Age range for inclusion was pre-pubertal*.

†† *Inclusive of prospective and retrospective enrollment*.

Table 2 overviews the diagnostic testing modalities of the individual studies according to PTHP disorders and summarizes time of injury to follow-up testing. A review of this table conveys that studies varied in both focus and duration, and suggests that guidelines for testing time windows in all TBI patients have not been universally adopted (44). Three of 15 studies included time points in the acute phase after TBI, with the remainder reporting follow-ups as far as 10 years post-injury. PTHP diagnoses can be delayed or missed due to testing intervals and are compounded by the transience of some disorders. Earlier testing and diagnosis may be helpful in making decisions on interventions. In addition to the sources of heterogeneity above, significant variations emerged from diverse definitions of endocrinopathies and multiple differing basal and stimulatory tests used across studies. In some disorders, the testing modalities varied widely (e.g., growth hormone deficiency) while in others they were similar (e.g., hypothyroidism). As previously reviewed, normal laboratory ranges commonly differed as well (7, 45).

**Table 2:** PTHP diagnostic approaches.

		**Testing modality**			
**References**	**Follow-up time post-injury**	**ACTH/cortisol**	**Thyroid function**	**GH**	**Gonadotropins**
Niederland et al. (29)	30.6 ± 8.3 mos	Basal cortisol ITT	Basal T_3_, T_4_, TSH TRH stim	Random GH L-DOPA ITT	–
Heather et al. (30)	6.5 ± 3.2 yrs	Basal cortisol Low dose ACTH stim. (1 μg), repeat if abn If failed ACTH stim.: metyrapone test	Basal T_3_, T_4_, TSH	IGF-1/IGFBP-3 Arginine-clonidine stim	GnRH stim
Bellone et al. (31)	1–9.1 yrs	8 a.m. cortisol, ACTHGlucagon stim	Basal T_3_, T_4_, TSH TRH stim	Bone age IGF-1 HV <3rd %ile at 6 mos or HV <25th %ile at 12 mos: GHRH-arginine stim	Plasma LH/FSH T, E_2_ LHRH stim
Auble et al. (32)	1.4–8.3 yrs*	8 a.m. cortisol Low dose ACTH stim. (1 μg/m^2^)	Basal T_4_ TSH surge	IGFBP-3 IGF-1 (>4 years and/or >15 kg) Overnight GH secretion	–
Einaudi et al. (33)	0–12 mos	8 a.m. ACTH and cortisol If abnl. ACTH, cortisol, or sx:glucagon stim	Basal T_3_, T_4_, TSH	IGF-1 HV <25th %ile and low/nl IGF1: GHRH-arginine HV <25th %ile and nl GH peak after GHRH-arginine: nocturnal spont. GH secretion	Bone age, Basal LH/FSH, T, E_2_ GnRH stim
Poomthavorn et al. (34)	0.9–8.5 yrs	AM cortisol If poor HV and low IGF1: glucagon stim	Basal T_4_, TSH	IGF-1/IGFBP-3 If poor HV and low IGF1: glucagon stim	LH/FSH, T, E_2_
Kaulfers et al. (35)	0–12 mos	8 a.m. cortisol 6 mos: low dose ACTH stim. (1 μg/m^2^)	Basal T_4_, TSH TSH surge	IGF-1/IGFBP-3 6 mos: nocturnal spont. GH secretion 12 mos if abn overnight GH secretion: arginine-clonidine or arginine-GHRH (age-dependent)	LH/FSH testing based on clinical signs
Srinivas et al. (36)	Days 0, 3, 7	8 a.m. cortisol, ACTH	Basal T_3_, T_4_, TSH	Random GH	–
Norwood et al. (37)	0.7–3.4 yrs^†^	AM cortisol	Basal T_4_, TSH	IGF-1/IGFBP-3 Nocturnal GH secretion Arginine-glucagon stim.	LH/FSH, T, E_2_
Khadr et al. (38)	1.4–7.8 yrs	AM cortisol, ITT or glucagon stim. if seizures	Basal TSH, T_4_	IGF-1 ITT or glucagon stim. if seizures	Low-dose GnRH stim.
Casano-Sancho et al. (39)	Months 3, 12	8 a.m. cortisol, glucagon stim.	Basal T_4_, TSH	IGF-1 Clonidine-glucagon stim	LH/FSH, T, E_2_ in pubertal patients GnRH stim
Personnier et al. (40)	9.5 ± 3.4 mos	8 a.m. cortisol	Basal T_3_, T_4_, TSH TRH stim. If abn	IGF-1 >15 kg: betaxolol-glucagon stim.; if low GH, arginine-insulin <15 kg or asthma: glucagon stim; if low GH, arginine	Pubertal patients: Plasma LH/FSH; Males >11 yrs or precocious puberty: T Females >10 yrs or precocious puberty: E_2_
Salomón-Estébanez et al. (41)	1.3–5.8 yrs	AM cortisol, ACTH If low cortisol, nl ACTH: ITT	Basal T_4_, TSH	IGF-1/IGFBP-3	Basal LH/FSH, T, E_2_
Dassa et al. (42)	5–10 yrs	8 a.m. cortisol, ACTH High dose ACTH stim. (250 μg)	Basal T_3_, T_4_, TSH	IGF-1 Arginine-insulin Glucagon-propanolol ITT Nocturnal GH secretion	Pubertal patients: Plasma LH/FSH; Males >11 yrs or precocious puberty: T Females >10 yrs or precocious puberty: E_2_ GnRH stim
Daskas et al. (43)	6.8–10.8 yrs	ITT	Basal T_4_, TSH	IGF-1/IGFBP-3 Nocturnal GH secretion ITT	Basal LH/FSH Basal T and E_2_

*Pre-pubertal and pubertal studies are represented in the first four rows*.

*Retrospective analyses not included*.

Mos, months; Yrs, years; Abnl., abnormal; Sx, symptoms; Nl., normal; HV, Height velocity; Stim., stimulation; Spont., spontaneous; T, testosterone; E_2_, Estradiol; ITT, Insulin Tolerance Test; N/A, Information not available; *Calculated using age at time of injury and age at the time of assessment. Mean years of both GHD and non-GHD groups.

Pediatric PTHP studies suffer from several irregularities which make deriving generalizable conclusions about the existence of sexual dimorphism difficult. Table 3 describes PTHP incidence or prevalence per study calculated with the total number of patients diagnosed with each disorder divided by either the total number of patients tested for that disorder or the total sample size, depending on the study and available data. The incidence or prevalence of each disorder varied widely across studies. While sex distribution was reported within the population of enrolled patients, frequencies of males and females tested and diagnosed for specific PTHP disorders was often missing. In Table 4, we attempt to discriminate incidence in males vs. females (again, subject to reporting of relevant information, and with denominators being total males or total females in the study). A discussion of what insights we can derive with respect to sexual dimorphism follows our review of individual disorders.

**Table 3:** PTHP incidence/prevalence by disorder.

**References**	**Sample size**	**% Central DI (# females/sample tested)**	**% ACTH deficiency/hypocortisolism (# females/sample tested)**	**% Non-thyroidal illness/central hypothyroidism (# females/sample tested)**	**% GHD (# females/sample tested)**	**% Central precocious puberty (# females/sample tested)**	**% Secondary hypogonadotropic hypogonadism (# females/sample tested)**
Niederland et al. (29)	26	0 (0/26)	34.6 (unk)	0 (0/26)	42.3 (3/26)	–	–
Heather et al. (30)	198	0.5 (1/198)^a^	0 (0/198)	0.5 (1/198)	0 (0/198)	1.0 (1/198)^a^	0 (0/198)
Bellone et al. (31)	70	0 (unk)	2.9 (unk)	1.4 (unk)	5.7 (unk)	1.4 (unk)	1.4 (unk)
Auble et al. (32)	14	0 (0/14)	0 (0/14)	33 (unk)	16.7 (unk)	–	–
Einaudi et al. (33)	52^b^	Acute: 3.3 *(1/30)*	Acute: 0 *(0/30)* Chronic: 5.9 *(0/34)*	Acute: 23.3 *(2/30)* Chronic: 2.9 *(0/34)*	Chronic: 10 *(0/30)*	Chronic: 2.9 *(0/34)*	Chronic: 2.9 *(0/34)*
Poomthavorn et al. (34)	33^b^	6.1^a^(0/33)	18.1^c^ (unk)	9.1 (0/33)	12.1 (1/33)	3.0 (1^a^/33)	6.1 (0/33)
Kaulfers et al. (35)	31^d^	Acute: 11.1 *(1/27)*	0 (0/*27*)	Acute: 7.4 (2/*27*) Chronic: 64 *(5/25)*	Chronic: 12.5 *(1/24)*	Chronic: 8.3 *(0/24)*	0 *(0/24)*
Srinivas et al. (36)	37^b^	–	0 (0/33)	0 (0/33)	0(0/33)	–	–
Norwood et al. (37)	32	3.1 (unk)	18.8 (unk)	0 (0/32)	15.6 (1/32)	0 (0/32)	12.5 (0/32)
Khadr et al. (38)	33	0 (0/33)	27.3 (1/33)	0 (0/33)	21.2 (0/33)	0 (0/33)	0 (0/33)
Casano-Sancho et al. (39)	37	0 (0/37)	43.5 (unk)	0 (0/37)	52 (unk)	0 (0/37)	0 (0/37)
Personnier et al. (40)	87	0 (0/87)	1.1 (0/87)	2.2^c^ (1/87)	81.8 *(7/33)*	0 (0/87)	0 (0/87)
Salomón-Estébanez et al. (41)	36	0 (0/36)	0 (0/36)	0 (0/36)	0 (0/36)	0 (0/36)	0 (0/36)
Dassa et al. (42)	66^d^	–	1.6 *(0/61)*	3.3 *(1/61)*	39.3 *(1^*e*^/61)*	6.6 *(3/61)*	0 *(0/61)*
Daskas et al. (43)	31^b^	0 (0/25)	8 (1/25)	0 (0/25)	24 (2/25)	0 (0/25)	4 (1/25)

*Pre- and pubertal studies are represented in the first four rows*.

*Sample tested—Represents either total sample size or total sample tested (when italicized)*.

*0–0%; UNK, Gender information not available*.

*–, Not tested; Acute, Deficiency detected during acute TBI phase*.

*Chronic, Deficiency detected during chronic TBI phase*.

a *Diagnosed prior to study procedures*.

b *Prospective and retrospective sample aggregates*.

c *Partial reports of sex information included*.

d *Sample size decreased over the study duration due to loss of follow up*.

e *1 of 6 patients with persistent GHD was female*.

**Table 4:** Pediatric PTHP disorders incidence by sex.

**References**	**Central DI (%)**	**ACTH deficiency/hypocortisolism (%)**	**Non-thyroidal illness/central hypothyroidism (%)**	**GHD (%)**	**Central precocious puberty (%)**	**Secondary hypogonadotropic hypogonadism (%)**
Niederland et al. (29)	M: 0/17 (0) F: 0/9 (0)	unk	M: 0/17 (0) F: 0/9 (0)	M: 8/17 (47.1) F: 3/9 (33.3)	–	–
Heather et al. (30)	M: 0/116 (0) F: 1/82 (1.2)	M: 0/116 (0) F: 0/82 (0)	M: 0/116 (0) F: 1/82 (1.2)	M: 0/116 (0) F: 0/82 (0)	M: 1/116 (0.8)^d^ F: 1/82 (1.2)	M: 0/116 (0) F: 0/82 (0)
Bellone et al. (31)	M: 0/58 (0) F: 0/12 (0)	unk	unk	unk	unk	unk
Auble et al. (32)	M: 0/11 (0) F: 0/3 (0)	M: 0/11 (0) F: 0/3 (0)	unk	unk	–	–
Einaudi et al. (33)^ab^	M: 0/27 (0) F: 1/7 (14.3)	M: 2/27 (7.4) F: 0/7 (0)	M: 6/27 (22.2) F: 2/7 (28.5)	M: 3/27 (11.1) F: 0/7 (0)	M: 1/27 (3.7) F: 0/7 (0)	M: 1/27 (3.7) F: 0/7 (0)
Poomthavorn et al. (34)^ab^	M: 2/21 (9.5) F: 0/12 (0)	unk^c^	M: 3/21 (14.3) F: 0/12 (0)	M: 3/21 (14.3)^d^ F: 1/12 (8.3)^d^	M: 0/21 (0) F: 1/12 (8.3)	M: 2/21 (9.5) F: 0/12 (0)
Kaulfers et al. ^a^ (35)	M: 2/18 (11.1) F: 1/13 (7.7)	M: 0/18 (0) F: 0/13 (0)	M: 12/18 (67) F: 5/13 (38.4)	M: 2/18 (11.1) F: 1/13 (7.7)	M: 2/18 (11.1) F: 0/13 (0)	M: 0/18 (0) F: 0/13 (0)
Srinivas et al. (36)	–	unk	unk	unk	–	–
Norwood et al. (37)	unk	unk	unk	M: 4/20 (20) F: 1/12 (8.3)	M: 0/20 (0) F: 0/12 (0)	M: 4/20 (20) F: 0/12 (0)
Khadr et al. (38)	M: 0/25 (0) F: 0/8 (0)	M: 8/25 (48) F: 1/8 (12.5)	M: 0/25 (0) F: 0/8 (0)	M: 7/25 (28) F: 0/8 (0)	M: 0/25 (0) F: 0/8 (0)	M: 0/25 (0) F: 0/8 (0)
Casano-Sancho et al. (39)	M: 0/30 (0) F: 0/7 (0)	unk	M: 0/30 (0) F: 0/7 (0)	unk	M: 0/30 (0) F: 0/7 (0)	M: 0/30 (0) F: 0/7 (0)
Personnier et al. (40)	M: 0/60 (0) F: 0/27 (0)	M: 1/60 (1.7) F: 0/27 (0)	unk^c^	M: 20/60 (33) F: 7/27 (25.9)	M: 0/60 (0) F: 0/27 (0)	M: 0/60 (0) F: 0/27 (0)
Salomón-Estébanez et al. (41)	M: 0/22 (0) F: 0/14 (0)	M: 0/22 (0) F: 0/14 (0)	unk	unk	M: 0/22 (0) F: 0/14 (0)	M: 0/22 (0) F: 0/14 (0)
Dassa et al. (42)	–	M: 1/49 (2) F: 0/17 (0)	M: 1/49 (2) F: 1/17 (5.9)	M: 5/49 (10.2) F: 1/17 (5.9)	M: 1/49 (2) F: 3/17 (17.6)	M: 0/49 (0) F: 0/17 (0)
Daskas et al. (43)	M: 0/20 (0) F: 0/11 (0)	M: 1/20 (5) F: 1/11 (9)	M: 0/20 (0) F: 0/11 (0)	M: 4/20 (20) F: 2/11 (18.2)	M: 0/20 (0) F: 0/11 (0)	M: 0/20 (0) F: 1/11 (9)
**Aggregate incidence**	M: 4/425 (0.7%) F: 3/205 (1.4%)	M: 13/348 (3.7%) F: 2/182 (1.1%)	M: 22/323 (22%) F: 9/166 (5.4%)	M: 56/373 (15%) F: 16/198 (8.1%)	M: 5/408 (1.2%) F: 5/210 (2.4%)	M: 7/408 (1.7%) F: 1/210 (0.5%)
**M:F Aggregate incidence ratio**	0.6	3.4	4.1	1.9	0.5	3.4

*Pre-pubertal and pubertal studies are represented in the first four rows*.

*M: _/_ (_%), total males positive for disorder/total males tested (calculated %)*.

*F: _/_ (_%), total females positive for disorder/total females tested (calculated %)*.

*unk, Information on sex not available; –, Not tested; Aggregate incidence, all positive males/all males OR all positive females/all females; M:F Aggregate incidence ratio, male aggregate incidence/female aggregate incidence*.

a *Prospective and retrospective cases aggregated*.

b *Total study sample used*.

c *Partial cases were reported without sex information; included in aggregate incidence if italicized*.

d *Diagnosed prior to study procedures*.

### Clinical Context of Post-Traumatic Hypopituitarism

Pediatric neuroendocrinopathies should be considered within the time course of TBI, which has traditionally been divided into acute and chronic phases. Acute TBI has been loosely defined as the first 2 weeks following the injury, though TBI-related neuroendocrinopathy studies have proposed durations which are shorter and longer (46, 47). Central diabetes insipidus (DI) is one of the most common acute PTHP disorders (48–50). Secondary adrenal insufficiency and non-thyroidal illness can occur in patients acutely as well (49, 51, 52). Vascular damage in the hypothalamus is the most likely explanation of acute neuroendocrine dysfunction after TBI (2).

Generally, the chronic phase after TBI is thought to begin weeks after the injury and can last well beyond 3 months (50). Although secondary adrenal insufficiency, central DI, and hypothyroidism can also occur in the chronic phase, other PTHP disorders emerge in this time frame—precocious puberty, hypogonadotropic hypogonadism, and growth hormone deficiency. The pubertal stages of patients are very relevant to the last 3 disorders. In the discussion below, we define pre-pubertal as ages 0–7 years, peri-pubertal as 8–13 years, and post-pubertal as 14–18 years.

Prospective studies have demonstrated that PTHP can be transient or persistent (35, 40, 53–57). Transient PTHP during both acute and chronic phases of TBI consists of episodes of hormonal deficiencies, the most recognized of which are acute-phase diabetes insipidus (DI) and secondary adrenal insufficiency. Prevalent management strategies such as the use of dopamine and etomidate can be one factor interfering with pituitary axes (58, 59). These typically resolve within the first few weeks of hospitalization.

The presentation of persistent PTHP can be delayed by months-to-years, which makes diagnosis highly dependent upon clinicians' indices of suspicion. Table 2 demonstrates inconsistent approaches to studying PTHP of delayed onset. Some studies enrolled patients years after their injuries to estimate a prevalence or incidence of persistent PTHP (29–34, 37, 38, 41–43). Longitudinal assessments over days to months captured cases of spontaneous resolution as well as persistent PTHP as remotely as 1 year post-injury (33, 35, 36, 39, 40), whereas cross-sectional assessments noted persistence and resolution up to several years after injury. In addition, prospective pediatric studies highlighted that attrition bias can emerge from losses to follow-up (35, 42). Therefore, like transient PTHP, the times of onset and resolution of the persistent PTHP disorders remain indeterminate as well.

Very little is understood about whether transient and persistent PTHP are induced by shared mechanisms, and it may be naïve to assume so. As mentioned, early PTHP during the acute phase of TBI may be the result of vascular insults such as infarction, infundibular disruption, and/or hypothalamic-pituitary suppressive medications (2–4). The transient stress of critical illness could also be involved in PTHP disorders like secondary adrenal insufficiency. Though episodes of early, transient PTHP disorders increase the likelihood of PTHP disorders with later onset (4, 55, 60), there is no evidence to suggest mechanistic overlap. The spreading of a proinflammatory response initiated by TBI and axonal injury that induces degenerative processes in distant brain regions has been offered as an explanation for the evolution of PTHP over time (61).

Very relevant to PTHP mechanisms and timing after injury during childhood but rarely a focus are sex differences in this constellation of neuroendocrine disorders. Differences in PTHP incidence between boys and girls are inconclusive when categorized by pubertal stage (Table 4), in part because of low numbers of cases. We separately discuss each PTHP disorder below.

### Central Diabetes Insipidus (DI)

Diabetes insipidus is typically diagnosed by a constellation of clinical symptoms and laboratory abnormalities consisting of progressive serum hypernatremia and excessively dilute polyuria. In our review, diagnostic criteria and testing modality did not markedly vary across studies (information not shown). Post-traumatic acute central DI had an all-severity incidence or prevalence of 0.5–11% in children (Table 3) and 15.4–51% in adults (48, 60, 62). Pediatric and adult studies of small sample size suggested that central DI is transient and associated with poor outcomes, yet resolves early in the acute phase of TBI; however, larger studies are needed to determine risk factors for rare instances of persistence (1, 35, 47, 48, 55, 60, 62). Central DI incidence did not appear to be a function of TBI severity (33, 35, 37). Most pediatric PTHP studies lacked sufficient data to suggest that central DI has a sex predilection, casting light on the need for larger studies.

### Secondary Adrenal Insufficiency

Three of our included studies (33, 35, 36) tested for secondary adrenal insufficiency in the acute phase after TBI. These employed basal ACTH and/or cortisol but found no cases in a total of 120 patients. The results from these pediatric studies contrast with adult studies which have reported an incidence of up to 78% in some series despite differences in testing (60, 63). Similar to central DI, data indicate adrenal insufficiency secondary to PTHP is TBI-severity independent (55), though it has been suggested otherwise (64). We surmise that the identification of early cases may be confounded by factors such as the ability to mount a hypothalamic-pituitary-adrenal (HPA) axis response following critical illness and medications received prior to testing.

Adrenal insufficiency in the chronic phase of TBI occurred at an incidence or prevalence of 0–43.5% in children (Table 3) and 4–19.2% in adults (54–56, 64, 65); the ranges are attributable, in part, to the variety of testing modalities employed. Most pediatric studies screened basal levels of ACTH and/or cortisol and confirmed adrenal insufficiency with cortisol stimulation (Table 2). A third of the reviewed studies used low or high-dose ACTH cortisol stimulation, while another third used insulin tolerance test (ITT) cortisol stimulation (Table 2). Three studies in our review did not use any cortisol stimulation testing (36, 37, 40). Dassa et al. identified one previously diagnosed male patient with ACTH deficiency using high-dose ACTH cortisol stimulation; the patient was treated 1 year post-injury and had resolution at 5.7 years (42). Kaulfers et al. did not use any basal ACTH measurements, which may not be necessary to make a secondary adrenal insufficiency diagnosis (35, 66, 67). In contrast, Niederland et al. (29), Salomón-Estébanez et al. (43), Khadr et al. (38), and Daskas et al. (41) used ITT as a cortisol stimulation test in children. Bellone et al. (34), Einaudi et al. (38), Poomthavorn et al. (31), and Khadr et al. (33) used glucagon stimulation. Heather et al. used metyrapone as a secondary cortisol stimulation test (30).

ITT is the gold standard for secondary adrenal insufficiency diagnosis. It is contraindicated for patients with histories of seizures and cardiac events, making it a higher risk test for some children and older adults (68–70). Some centers, in fact, do not offer it. Metyrapone is less commonly used, as it is difficult to obtain, requires overnight observation, and poses a risk of adrenal crisis (71, 72). ACTH cortisol stimulation is more sensitive, rapid, and safe than both ITT and metyrapone, but it is not as specific (68, 70, 71, 73). The diagnostic thresholds with ACTH stimulation are more clearly defined for primary adrenal insufficiency but are less clear for secondary (68, 72).

These differences in safety, accuracy, and logistics not only explain the wide range of diagnostic incidence but also indicate the need for consensus testing guidelines.

Although pediatric studies reported male and female enrollment, they did not explore sexual differences in secondary adrenal insufficiency by pubertal stage (Tables 3, 4). Adult studies have also not made direct sex comparisons of prevalence (54–57, 64, 65, 74). The time to resolution is also unknown. Some adult studies have reported prevalences that tend to decrease over time—for example, from 8.5 to 7.1% between 3 and 12 months post-injury in one study (57) and 20 to 6.6% between 1 year and 3 years post-injury in another study (56). Overall, the data indicates that larger and more granular longitudinal studies are needed, especially in children.

### Acute Non-Thyroidal Illness and Late Hypothyroidism

Following TBI and other critical illnesses, circulating thyroxine (free or total T_4_) may not be converted to tri-iodothyronine (free or total T_3_), reducing the amount of circulating T_3_ available and leading to low T_3_ syndrome, otherwise known as non-thyroidal illness (75). In addition, non-thyroidal illness is also characterized by low-to-normal T_4_ and normal TSH (75).

In contrast to the other PTHP disorders, pediatric studies of post-traumatic thyroid dysfunction have been relatively consistent in the diagnostic testing methods employed, measuring basal T_4_ and TSH, with or without the use of T_3_ (Table 2). Two pediatric studies measured T_3_ during the acute phase of TBI: Srinivas et al., who did not diagnose any cases of acute non-thyroidal illness, and Einaudi et al., who reported an incidence of 23% (Table 3) (33, 36). Kaulfers et al. did not use basal T_3_ and found an incidence of 7.4% (Table 3) (35). The reported prevalence of acute non-thyroidal illness following TBI in adults was generally higher, ranging from 33 to 51% (54, 55, 64). Sex differences in the occurrence of acute non-thyroidal illness following TBI have not been elucidated in adults or children.

Overall, the incidence or prevalence of hypothyroidism reported in the chronic phase of TBI is 0–64% in children (Table 3) and 1–44.3% in adults (2, 46, 54–57, 74). In chronic phase studies of pediatric TBI, some used confirmatory testing in addition to the standard thyroid panel, including TSH stimulation and measurements of TSH surge. TSH surge is a nocturnal increase over the mid-afternoon to early morning hours, that can be screened for by two blood samples at 8 a.m. and 4 p.m. (51, 76). Two studies employed TSH surge assessments: Kaulfers et al. measured TSH surge at 6 and 12-months following TBI, whereas Auble et al. tested years following the injury (32, 35). Incidences were 64 and 33%, respectively. Notably only 2 patients were treated based on abnormal TSH surge testing in the Kaulfers et al. study (35). TRH stimulation with serum TSH measured at baseline and after stimulation was used by Niederland et al. but failed to show differences between TBI and control groups (29). Personnier et al. and Bellone et al. used TRH stimulation as well with small incidences, 2.2 and 1.4% respectively (31, 40). Adult TBI studies assessing central hypothyroidism used basal morning samples with no stimulation testing or TSH surge measurements (55, 74, 77).

Central hypothyroidism testing during chronic TBI has not yielded compelling evidence for sex differences in children or adults. Kaulfers et al. reported the greatest number of cases in our pediatric studies, diagnosing 12/18 males (67%) and 5/13 females (38.4%) (Table 4). Though the majority of cases (all but 2) resolved before 12 months, the authors concluded that peri-pubertal males were the most susceptible to developing central hypothyroidism at the 6–8 month interval followed by post-pubertal females (35). While this study of 31 subjects suggested a difference between male and female incidence of transient, chronic hypothyroidism, the other studies we reviewed did not provide sufficient data to support a conclusion about sexual dimorphism.

### Central Precocious Puberty

Isosexual central precocious puberty (precocious puberty) is due to early and increased gonadotropin-releasing hormone (GnRH) secretion. Generally speaking, sexual precocity is defined by both the timepoints of development and progression over time. Precocious puberty in TBI patients has an overall incidence or prevalence of 0–8.3% (Table 3). Pubertal stages were assessed in studies of precocious puberty primarily using Tanner staging (30, 31, 33–35, 37–43). Occasionally, menstrual history or Tanner stage 2 development in girls ≤ age 8 years and testicular volume > 4 mL in boys before age 9 or 9.5 years was used.

Laboratory assessments of precocious puberty were variable. About two-thirds of the studies we reviewed measured basal gonadotropins in the chronic phase of TBI with or without estradiol or testosterone measurements (Table 2). Estradiol and testosterone were assayed in a total of nine studies, all of which included either pubertal or post-pubertal stage and/or clinical signs of puberty. Fluctuations can occur with basal serum hormone concentrations in the diagnosis of precocious puberty, and GnRH stimulation has been shown to be sensitive and specific to make a central precocious puberty diagnosis (78). Five studies performed GnRH stimulation and one stimulated with LHRH. There were also variations in assays used to assess basal LH, FSH, estradiol, and testosterone concentrations, i.e., radioimmunoassays and immunofluorescent assays. More information is needed on best testing practices for this disorder.

There was also temporal variation in how pediatric studies assessed precocious puberty. In Kaulfers et al., the incidence of precocious puberty increased 6–8 months post-injury (35), suggesting it may be appropriate to begin screening by physical exam in girls < 8 years and boys < 9 years during the early chronic phase. This is also supported by the observation that of all cases, retrospective review identified 3 of 11 previously diagnosed cases, years following the injury (30, 33, 34). Studies that identified prospective cases years following TBI (up to 6.1 years in the Dassa et al. study) suggest this PTHP disorder may persist or have delayed onset (42).

In order to contextualize the occurrence of precocious puberty after TBI, it is important to understand that central precocious puberty has an estimated general population prevalence of 8 in 10,000 in girls and 1 in 10,000 in boys (79). The reported incidence or prevalence of precocious puberty in pediatric PTHP studies was variable but low (Table 3), and no direct sex differences were assessed. Heather et al. reported that the precocious puberty prevalence for girls in their study (1.2%; *n* = 1/82) did not differ from the general population, whereas their data represented a higher than expected prevalence in boys (0.8%; *n* = 1/116) (30). Across studies, precocious puberty was identified in 11 patients, and girls had a 2-times higher incidence or prevalence than boys [Table 4—the sex of one case was not reported (31)]. Dassa et al. identified 17.6% of female participants (*n* = 3/66) with precocious puberty, of which 33% also had growth hormone deficiency (GHD) (1 female) (42). Therefore, individual studies cannot offer conclusions about the existence of sexual dimorphism in post-traumatic precocious puberty. There are no known adult studies reviewing cases of precocious puberty in childhood following a TBI.

### Secondary Hypogonadotropic Hypogonadism

Post-traumatic hypogonadotropic hypogonadism leads to delayed puberty in children and decreased quality of life in adults. The overall incidence reported by pediatric PTHP studies is 0–12.5% (Table 3). The sex of one case was not reported (31). Though it has been understudied using small sample sizes in children, reports of post-traumatic hypogonadotropic hypogonadism are likely rare because peripubertal children must be studied longitudinally in order to observe the severity of pubertal failure. As mentioned, Daskas et al. prospectively identified one female with secondary hypogonadotropic hypogonadism who also had abnormal GH secretion (43). Conversely, Norwood et al. reported that 100% of males (4/4) with growth hormone deficiency (GHD) had lower FSH and lower testosterone levels (37) in comparison to non-GHD patients, yet, these participants were not identified as being at risk for secondary hypogonadotropic hypogonadism. Testosterone assay types and the testing times of day may cause over- or underestimation of testosterone levels (80). Recent studies in children with reports of secondary hypogonadotropic hypogonadism have relied on mixed retrospective-prospective review rather than longitudinal testing (33, 34). Three male cases of secondary hypogonadotropic hypogonadism were retrospectively reported by Einaudi et al. (33) and Poomthavorn et al. (34) but no cases were found prospectively. The data from these individual studies are not sufficient to reach conclusions about sexual dimorphism in post-traumatic hypogonadotropic hypogonadism.

### Growth Hormone (GH) Axis Abnormalities

Somatotrophs (GH secreting cells) make up a large portion of the anterior pituitary and are situated laterally within the gland, prompting some to propose that there is an increased risk of injury to these cells after TBI. Both somatotrophs and gonadotrophs (LH/FSH secreting cells) are supplied by the long hypophyseal artery, which originates from above the sella and is susceptible to injury (4).

Growth hormone deficiency (GHD) is one of the most common anterior pituitary abnormalities in PTHP. Pediatric and adult incidences or prevalences range from 0 to 82% (Table 3) and 10.7–43.3% (55–57, 64, 74), respectively, which can be influenced by testing modalities (1, 35). The pediatric studies we reviewed used auxological measurements along with laboratory testing. Pediatric studies have employed a number of laboratory screening and confirmatory testing modalities for the assessment of post-TBI GHD, which are summarized in Table 2.

In most studies, IGF-1 and IGFBP-3 screening guided the use of confirmatory testing and provided supportive evidence of a GHD diagnosis. Salomón-Estébanez et al. used IGF-1 and IGFBP-3 to identify children with potential GHD but did not observe GH abnormalities that warranted further testing (41). Basal GH screening alone was used in one acute hypopituitarism study (36), however, this testing modality is not typically used to diagnose GHD. Six studies also supported the use of confirmatory testing by measuring spontaneous nocturnal GH levels (32, 33, 35, 37, 42, 43). Auble et al. used spontaneous nocturnal GH testing to support later GH stimulatory testing outside of study procedures (32).

Following IGF-1 ± IGFBP-3 screening, GHD diagnoses were confirmed using one or more stimulatory tests, and all studies used different testing cutoffs. The insulin tolerance test (ITT) was used most frequently (in 4 of 15 studies; see Table 2). IGF-1 and IGFBP-3 screening results did not always correlate with those of the ITT-stimulation test, though they did with spontaneous nocturnal GH secretion (43). It has been previously reported that IGF-1 and IGFBP-3 testing is not as reliable as ITT-stimulation in the diagnosis of GHD in adults (81, 82). Furthermore, ITT was used as a confirmatory test in different ways: (1) alone in patients without seizures (diagnostic GH cutoff <5 ng/mL) (38); (2) in combination with L-DOPA which assesses GH reserve [cutoff 0.07 ng/mL (7 ng/dL)] (29); (3) as part of a testing panel (cutoff <7 ng/mL) (42); and (4) following spontaneous nocturnal GH measurements (age-normalized cutoffs of <3 to <6.7 ng/mL (43).

Both Heather et al. and Kaulfers et al. used arginine-clonidine GH stimulation following IGF-1 and IGFBP-3 screening (30, 35). GHD diagnostic cutoffs ranged from <5 ng/mL to mean spontaneous nocturnal GH below the lower 95% confidence limit for age and pubertal stage (30, 35). IGF-1 and IGFBP-3 levels did not correspond to GH testing in the longitudinal study by Kaulfers et al. (35).

Other types of stimulatory testing for post-TBI GHD included arginine (40), GHRH-arginine (31, 33, 35), arginine-insulin (40, 42), glucagon-propanolol (42), betaxolol-glucagon (40) clonidine-glucagon (39), glucagon (34, 38, 40), and glucagon-arginine (37). The majority of these studies were used as primary confirmatory GH tests or as part of a testing panel, all with different diagnostic cutoff values. In one study, GHRH-arginine detected GHD in conjunction with abnormal height velocity using a cutoff of <20 ng/mL (33). Not surprisingly, the range of testing approaches resulted in a varied incidence or prevalence (Table 3).

Some studies have evaluated partial and complete GHD. Personnier et al. looked at 87 children 6–18 months post-injury using a complex testing algorithm: partial GHD was defined as 5–7 ng/mL and complete GHD was <5 ng/mL (40). Two confirmatory testing panels were used—the primary panel was betaxolol-glucagon (children ≥15 kg) or glucagon (<15 kg or asthma), and the secondary was arginine-insulin (children ≥15 kg) or arginine (children <15 kg). Partial or complete GHD diagnosis was made using peak GH <7 ng/mL with 2 confirmatory tests. Of 87 patients, 12 were found to have partial GHD (13.8%) and 15 complete GHD (17.2%) (40). The Personnier et al. study was followed by a longer investigation by Dassa et al. with the same study cohort (40, 42). Dassa et al. used another testing panel (arginine-insulin, glucagon-propanolol, ITT, with spontaneous GH testing) with a diagnostic cutoff of <7 ng/mL using 2 confirmatory tests (42). Adult studies have also evaluated partial and complete GHD using different confirmatory values with a prevalence of 15.7% partial and 22.8% complete GHD (57).

GH levels are influenced by concentrations of sex steroids, which increase as puberty progresses (83). Therefore, in children nearing puberty, sex steroids are used to maximize the response of GH during stimulatory testing (84). We found that sex steroid priming was not always performed prior to GH testing. Sex steroid priming and increased body-mass index (BMI) can influence GH stimulation testing results, adding further complexity to assessments. Thus, pubertal stage also plays a significant part in GH evaluation. TBI can induce abnormalities in the GH axis directly as well as indirectly, via pubertal perturbation, providing additional reasons why children should be cohorted based upon pubertal stage in order to study sexual dimorphisms. No sex differences were reported in the adult studies we reviewed (54, 74).

These studies convey the complexity of making a GHD diagnosis. There was major variability in basal and stimulatory testing with age-related reference ranges. There was often poor correlation among testing regimens and between auxological measurements and testing results. Finally, assessments were done at various time points during the course of TBI recovery. These inconsistencies provide an example of the broad need for standardization of pituitary hormone testing and measurements during the time of recovery and rehabilitation.

### Sexual Dimorphism Within PTHP Disorders

From the discussion above, it is evident that studies of pediatric PTHP suffer from several irregularities which make deriving generalizable conclusions about sexual dimorphism difficult. While studies often reported the sex distribution within the population of enrolled patients, information on how many males and females comprised the subpopulations tested for specific disorders was frequently missing (indicated in Table 4 as “unk”). From the data we could extract and collate, we first calculated the aggregate incidence by sex for each PTHP disorder by adding cases and sex-specific sample sizes for each disorder across studies, and then generated male-to-female aggregate incidence ratios (M:F AIRs) by dividing male incidence by female incidence. We understand prevalence and incidence of each study are calculated differently and our calculation may not be ideal considering this point. This metric attempts to correct for the universal male predominance in enrollment; however, when female sample sizes are small (<20 in some studies), the aggregate PTHP disorder incidence becomes more subject to chance. Also, one would have to assume a degree of equivalency in the diagnostic testing employed across studies—as well as in males vs. females—to consider these M:F AIRs to reflect true degrees of sexual dimorphism. This assumption is belied by wide variations of disorders even in males, for example, 12/18 cases of central hypothyroidism in Kaulfers et al. but 0/116 case in Heather et al. That said, the M:F AIRs we derive represent the best possible estimation of sexual dimorphism in pediatric PTHP disorders at this time, and may provide a basis for hypotheses which can be tested in future studies.

Of the PTHP disorders in children, all except central DI and central precocious puberty appear more likely to occur in males vs. females. The highest male PTHP predilection of 4.1 is in central hypothyroidism, which is derived from only 31 total cases across all studies with sex information reported; 9 cases out of 276 patients were not included due to lack of information about sex (studies labeled as “unk” in Table 4). ACTH deficiency and secondary hypogonadotropic hypogonadism each appear to be over 3 times more likely in males, derived from 15 cases (33 cases out of 235 patients not included) and 8 cases (1 case out of 70 patients not included), respectively. Central DI and central precocious puberty each seem twice as likely to occur in females based upon 7 cases (1 case out of 32 patients not included) and 10 cases (1 case out of 70 patients not included), respectively. The low case number of central DI is striking when compared to adult reports. However, clinical experience suggests that central DI is among the most common acute PTHP disorders (49) though its occurrence after TBI may also portend death (60, 85) and likely precludes it from most PTHP studies which focus on the chronic phase. The estimated AIR of 0.5 for central precocious puberty included 10 cases. Again, the female predilection suggested by the AIR of precocious puberty should be interpreted carefully—while the aggregate incidence in females was 2 times higher, the male rate is likely more significantly increased compared to what it is in the general population. GHD is 2 times more likely to occur in males than females based on 72 cases (18 cases out of 194 patients not included). Each of these pediatric PTHP disorders requires larger, comparable study designs as well as standardized diagnostic testing approaches, to derive accurate insight into sexual dimorphism.

## Reciprocal Influences Among Common TBI and PTHP-Related Consequences

We have discussed how several features of existing studies, including male-to-female ratios of TBI populations and a mixture of pubertal stages, make sex differences in PTHP difficult to discern. In this section, we assume two new vantage points. From one, we discuss a set of complications—sleep disorders, neuropsychological disturbance, and cognitive dysfunction—which can equally be worsened or caused by PTHP and TBI and exhibit sexual dimorphisms of their own. The distinction is significant: for example, it would suggest a male victim of TBI with depression may need a hypothyroid state excluded before being treated with antidepressants. From a second vantage point, we discuss how adverse childhood events (ACEs) may confound outcomes after PTHP and TBI in a potentially sexually dimorphic way.

### Sleep Disorders

Up to 70% of patients experience sleep disorders after TBI in a manner that is independent of injury severity (86). These disorders can include insomnia, circadian rhythm disturbances, and sleep apnea. When compared to children sustaining orthopedic injuries sparing the head, preschool children with TBI exhibited reduced sleep duration and bedtime resistance (87). In children, those with preexisting conditions such as ADHD prior to TBI were found to have higher rates of sleep disturbance (88). Women seem to have a higher incidence of sleep disorders after one incidence of mild TBI; however, with recurrent injuries, both males and females report sleep disorders equally (89). Pre-injury co-morbidities, such as headache or migraine, seem to increase risk of post-injury sleep disorders in adults (90). Some of these co-morbidities may be expressed in sexually dimorphic patterns, and this likely impacts the sleep disorder incidence post-TBI based on sex.

Sleep is also intimately and reciprocally tied to several pituitary axes, even without TBI. In a case-control study of adults with GHD, sleep quality, daytime sleepiness and sleep –wake cycles were disturbed in the GHD group vs. the control group, irrespective of GHD etiology (i.e., pure pituitary, pituitary with possible hypothalamic involvement, idiopathic childhood onset, hypothalamic) (91). While hypothyroidism is associated with abnormal ventilatory drive, abnormal sleep architecture, and sleep apnea in adults, less is known about its effects in children (92). Conversely, disruption of sleep can dysregulate gonadotropin release during puberty and surges in TH and GH at all stages of life, potentially impacting PTHP-related outcomes such as linear growth.

Zhou et al. found that in adults, mild TBI-related HPA axis dysfunction (exhibited by low cortisol following ACTH stimulation) was associated with insomnia when compared to a control group (93). Unfortunately, sex-related variables were not reported. Additional studies are needed in both children and adults to elucidate the influence of PTHP in post-traumatic sleep disorders and vice versa, as well as the degrees to which females and males are differentially affected. Attention to pre-existing conditions may also be important.

### Neuropsychological Disturbances

Individuals with TBI show significantly elevated rates of depressive and anxiety disorders, most commonly major depressive disorder and PTSD, which are most likely to emerge in the first year post-injury. In adults, female sex has been associated with increased risk of anxiety and mood disorders in some studies of TBI but not others (94, 95). A post-TBI study found that male sex increased risk of post-injury substance use disorder (96). Perimenopausal women have an increased susceptibility to psychiatric disorders following TBI such as depression and anxiety (28). Although there are many aspects predisposing patients to psychiatric disorders, reasons for sexually dimorphic psychiatric responses remain unclear. One possible explanation for perimenopausal women having more susceptibility than men to TBI-related psychiatric disorders is that women have increased neuroinflammatory responses to injury stress (97). Increased neuroinflammation may be plausible although the contribution of hormonal changes during perimenopause cannot be discounted.

Symptoms of PTHP-related hormone deficiencies—particularly hypothyroidism, hypogonadism, and GHD—may masquerade as TBI-related neuropsychological symptoms such as PTSD and depression (98). Neuropsychological dysfunction occurs in patients with overt hypothyroidism and can improve with thyroid hormone replacement. Mild hypothyroidism typically is not the cause of significant neuropsychological symptoms (99). Children with GHD may express higher levels of anxiety than controls, with untreated patients exhibiting the highest levels (100). Perhaps early androgen exposure can cause some neurological changes that facilitate development of neuropsychological conditions. Testosterone concentrations are proposed to alter limbic and hippocampal structures as evidenced by a study using fMRI in boys with familial male limited precocious puberty (101). Females with earlier androgen exposure have higher rates of oppositional defiant disorder, and higher symptom counts reflecting anxiety, mood, or disruptive behavior disorders (102). Without targeted evaluations, it can be difficult to discriminate PTHP or TBI as the principle contributor to a newly recognized post-TBI neuropsychological disturbance.

Neuropsychological disturbances are also associated with sleep disorders as well as TBI and the hormonal changes observed in PTHP. In a national sample of 11,670 U.S. participants (5,594 females, aged 9–10 years, 63.5% white) in the Adolescent Brain Cognitive Development study, sleep disturbances co-varied with development of future mental health issues, particularly depression (103). Specific disturbances such as depression, anxiety, post-traumatic stress disorder (PTSD), substance abuse, and psychoses are closely associated with TBI, regardless of severity (104) and thus may manifest in even mild TBI.

Sexual dimorphism, and even an age effect, is noted with neuropsychological symptoms. Yue et al. reported women of ages 30–39 years with mild TBI had increased PTSD episodes 6-months post-injury when compared to men of ages 30–39 years as well as younger women and men of ages 18–29 years (28). Another study by Lavoie et al. failed to show a statistically significant difference between men and women in reports of neuropsychological sequelae, again, likely due to the disproportionate male-to-female ratio of participants affected by TBI (105). Based on this information, neuropsychological evaluations of TBI patients would benefit greatly from concomitant investigations of endocrine dysfunction, and studies should consider age, pubertal stage, the co-existence of PTHP, and sex differences.

### Neurocognitive Dysfunction

Neurocognitive dysfunction is well-described in adult TBI, with both acute (confusion, poor memory) and chronic (post-concussive syndrome) manifestations. Studies in children are not common but suggest that children report worse cognitive symptoms 1 year post-concussion than do adults (106). Deficits in executive function (107) as well as disruption of cognitive development and decreases in acquisition of new knowledge have been noted (108). Furthermore, it is suspected that children may experience post-injury cognitive dysfunction in a sexually dimorphic way (109). In a controlled study of 70 all-severity TBI patients between ages 6 and 16 years, Donders et al. reported memory dysfunction due to decreased information processing speed in boys compared to “demographically-matched” controls and girls post-injury (110).

We know that post-traumatic attention deficits, memory impairment, and alterations in information processing speed, language, and visuospatial skills, can overlap with sequelae of PTHP-related hormone deficiencies in adults (111). In a study of 72 adult TBI patients ages 17–73 years (56 men, 16 women), PTHP, particularly GHD and hypogonadism, were associated with decreased cognitive functioning along with decreased functional independence and increased disability ratings (77). A correlation between GHD and fatigue and depression but not cognitive dysfunction was observed in children, adolescents, and young adults with TBI (43).

The relationship of PTHP to neurocognitive deficiencies has not been evaluated in children, and its study is particularly challenging as cognition in childhood is also influenced by extrinsic factors as we discuss in the next section. Non-traumatic neuroendocrinopathies lend some insight. Children with genetic panhypopituitarism have been reported to have learning difficulties in some cases (112), though the evidence for any one pituitary hormone deficiency causing neurocognitive dysfunction is sparse and seems to be related to when the condition was acquired. Untreated congenital hypothyroidism causes severe intellectual and developmental delays, but mild subclinical hypothyroidism in childhood might not have the same effect (113). Children who are small for gestational age and receive growth hormone treatment may experience an improvement in indicators of neurocognitive function (114) while children who present with growth hormone deficiency later in life may not reliably show similar gains after treatment. On the other hand, in children with Prader Willi syndrome, GH replacement therapy prevented loss of certain skills related to cognition in the short term and strongly improved abstract reasoning and visuospatial skills over a period of 4 years (115). Adrenal insufficiency and diabetes insipidus would not be suspected to directly affect neurocognition, but rather have indirect effects based on the child's health status as a result of these conditions. Finally, pubertal development is expected to have significant effects on affective and motivational functioning (116) and it appears that changes in the usual pubertal tempo may have lasting effects on neurocognition (117).

Sex differences in neurocognition post-TBI have been studied but not as related to PTHP. In general, sex-specific hormone therapy in adults may benefit neurocognitive dysfunction. Adult male patients, for example, saw improvements in memory with testosterone replacement, and females saw improvement in verbal response with estradiol replacement (118–120). Perhaps timely identification and treatment of PTHP could improve neurocognitive outcomes in children and young adults during periods of development and/or recovery and prevent negative neurodevelopmental trajectories.

### Adverse Childhood Events and Impact of Socioeconomic Factors

Adverse childhood events (ACEs) are potentially recurring circumstances that place severe social, psychological, and physiological stress on a child in a way that is negatively consequential (121). ACEs include sexual, emotional, and physical abuse; emotional and physical neglect; mental illness; criminal activity; parental absence; domestic violence; substance abuse; school and community violence; extreme economic adversity; and other events that cause a child to experience extreme sense of danger or harm. Prevalence estimates suggest at least one ACE is present in 62% of the general population, but 25% of the population has three or more. Higher scores are observed in particular sub-populations—Black or Hispanic, lower socioeconomic status, and those identifying as bisexual/gay/lesbian (122). ACEs undoubtedly place adults and children in a higher risk category for suffering TBI, with greater odds of TBI if 3 or more ACEs are reported (123). In particular, increased parental stressors in children who have premorbid cognitive dysfunction and learning disabilities together increase TBI risk in children (124).

**Figure 1:**
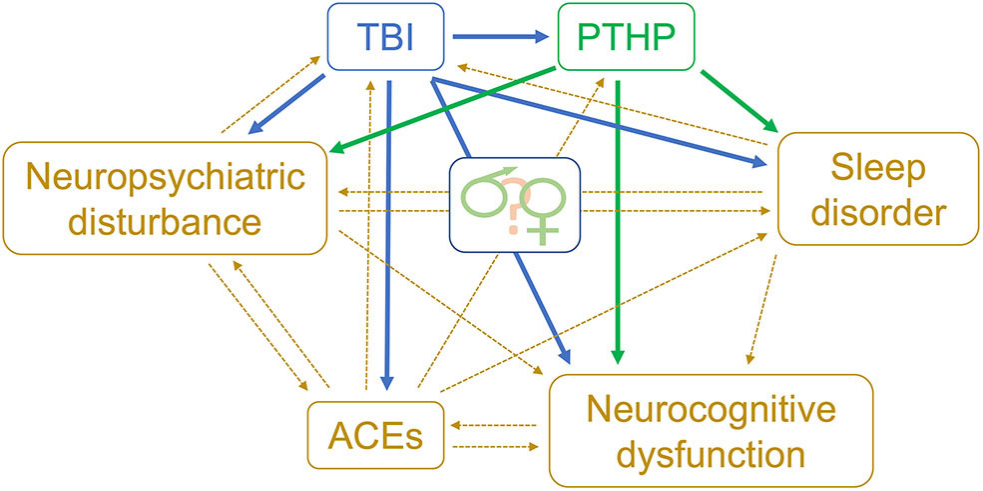
Biopsychosocial model of risk factors for TBI & PTHP. Theoretical model for relationships between adverse childhood events and PTHP. TBI, traumatic brain injury; PTHP, post-traumatic hypopituitarism; ACEs, adverse childhood events.

There are two plausible pathways by which ACEs could relate to the occurrence of PTHP (Figure 1). The first is when ACEs are followed by TBI. There are multiple reports of early life stressors altering HPA axis function via epigenetic mechanisms (125–128), and these changes may be modulated by sex as well as by the developmental stage at which the ACEs are suffered (129). Epigenetic mechanisms also underlie the impact of ACEs on immune responsiveness (130). ACEs tend to be recurring, leading to traumatic, toxic stress and possible chronic dysregulation of the HPA axis and a chronic immune response (131). Thus, individuals with significant ACEs may have pre-existing pituitary dysfunction and an altered inflammatory response to subsequent TBI, conceivably lowering the threshold for development of PTHP.

The second pathway by which ACEs could relate to the occurrence of PTHP is when TBI is followed by ACEs. Childhood TBI can also change stress responsiveness by epigenetic mechanisms (127, 132). Furthermore, psychiatric co-morbidities and PTSD are both known sequelae of TBI (133, 134) and both are associated with alterations in the HPA axis by similar mechanisms (135, 136). Many new stressors are introduced in a household following TBI, including altered family dynamics, economic hardship, shattered dreams, and an uncertain future due to challenges re-entering school or work programs (137–142). Such conditions could further increase the likelihood of ACEs, but may also increase the impact of those ACEs on neuroendocrine function that is more susceptible, especially involving the HPA axis.

Regardless of whether ACEs precede or follow TBI, what remains unclear but plausible is whether the consequences of ACEs on the HPA axis and immune function alter the risk of PTHP. With the high prevalence of ACEs, especially within disadvantaged populations, this is an important question to answer. Furthermore, given that (a) women tend to have higher mean ACE scores than men (122), (b) epigenetic consequences of ACEs may vary with sex as mentioned above, and (c) ACEs and socioeconomic factors are asymmetrically distributed across the sexes, ACEs may emerge as a sexually dimorphic risk factor for PTHP.

Biopsychosocial models which incorporate ACEs as well as sleep disorders, neuropsychological disturbances, and neurocognitive dysfunction, should also be considered to understand the contributions of interconnected factors in the sequelae of PTHP (Figure 1).

## Conclusions and the Path Forward

We initiated this review with the goal of describing sexual dimorphism in pediatric post-traumatic hypopituitarism (PTHP). We find and describe several reasons why the current state of the literature limits our ability to draw generalizable conclusions. (1) The inherently increased male-to-female ratio in TBI populations makes single-center subgroups too small for statistical comparisons by sex. (2) Studies enrolling children do not attempt to match pubertal stage. (3) Testing strategies and diagnostic criteria for each endocrinopathy vary widely. (4) A lack of guidelines for testing windows may result in missed diagnoses of transient and persistent PTHP as well as delayed intervention. (5) Single-center studies lack generalizability. (6) Losses in follow-up introduce attrition bias, which is a particular problem for pediatric studies. (7) Many studies lack control populations. (8) Numbers of male and female participants who develop or do not develop endocrinopathies are not reliably reported within studies.

Analogous problems have perpetually plagued the field of TBI until recently. To overcome them, in 2009 the National Institute of Neurological Disorders and Stroke (NINDS) launched the common data elements (CDE) project for standardized data collection across TBI studies (https://www.commondataelements.ninds.nih.gov). CDEs for adult TBI have recently been defined (Version 1.0) and are being developed for pediatric TBI as well (143–145). Already, multicenter studies enrolling TBI patients worldwide (e.g., ADAPT, TRACK-TBI) (143, 146–148) are collecting core datasets which allow for more direct comparisons of study differences, analyses of patients across studies, and the assembly of larger, common study populations. This presents an opportunity for neuroendocrine-related CDEs to be developed and included for pediatric and adult TBI.

**Figure 2:**
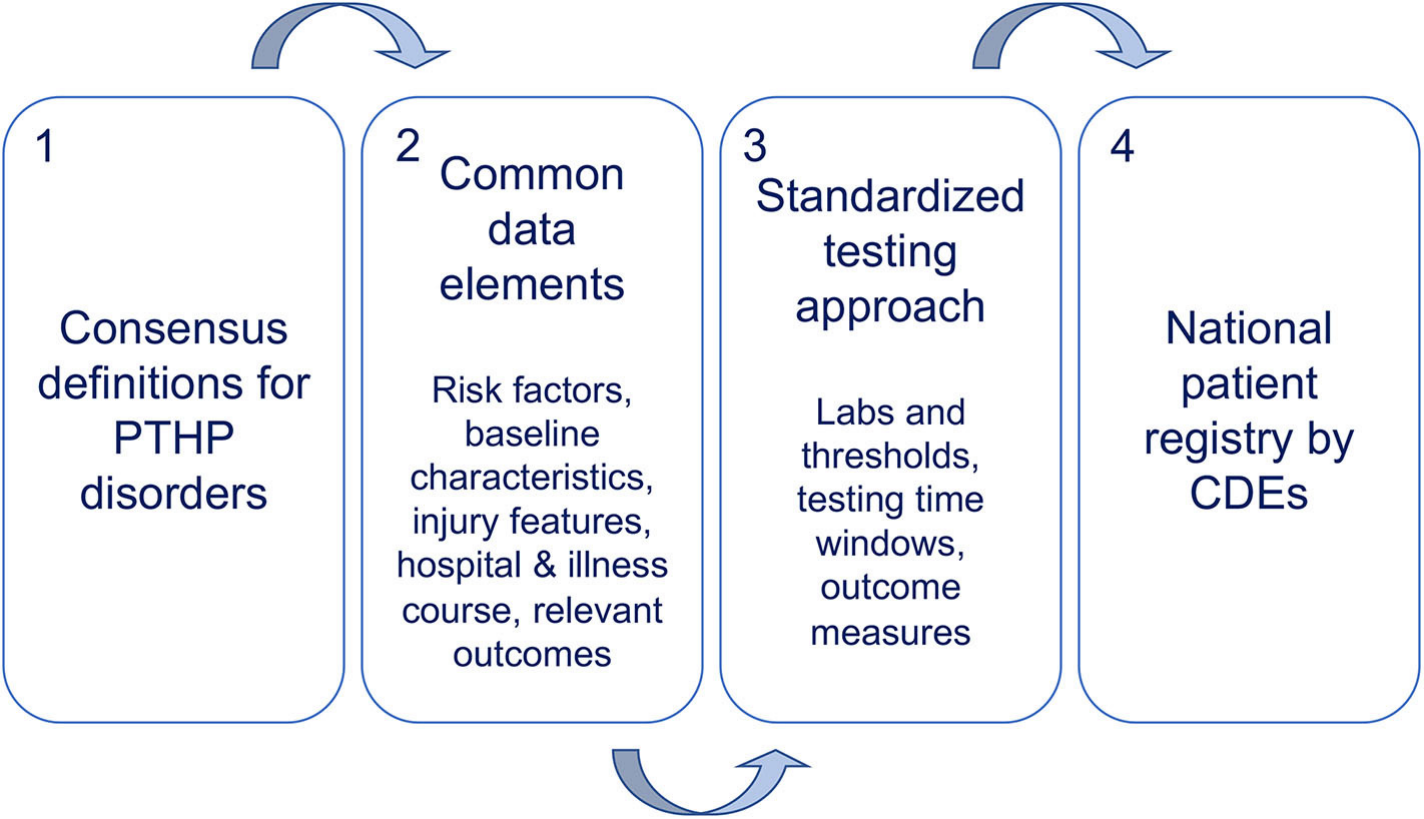
The path forward in the study of PTHP.

The study of PTHP would benefit immensely from the use of CDEs (Figure 2). First, investigators in the field would need to reach consensus on neuroendocrinopathy definitions that consider all stages of development. Second, investigators would identify CDEs that thoroughly describe patients in terms of risk factors and baseline characteristics, injury features, hospital and illness course, and relevant outcomes across primary and secondary domains of interest. We suggest these domains include endocrine, metabolic/nutritional, psychological, and cognitive features at a minimum. With respect to injury features, TBI CDEs could be employed to offer some alignment of these datasets, and more specific descriptors of pituitary injury could also be developed. Doing so would also allow better matching of injury types. Third, investigators would need to agree upon a standardized testing approach that encompasses laboratory evaluations, testing windows, and outcomes measurements. We are currently in the process of developing a standard testing framework for diagnosing PTHP across developmental stages which could serve as a point of embarkment. Positioning the field to have a patient registry defined by CDEs would also benefit ancillary studies in areas such as sleep disturbances, neuropsychology, cognition, and emerging research areas of TBI such as post-ICU care syndrome, metabolism, and ACEs along with social determinants of health.

Ideally, there should be a joint discussion among the pediatric TBI and endocrine communities of investigators. The best way to move forward is together: we propose a consensus working group to issue guidelines for these studies and to create a common, collaborative framework for prospective data collection.

## Author Contributions

AW, AD-T, and NS are responsible for conducting the literature search, writing, and editing all drafts of this manuscript. AD-T and NS contributed equally to the composition of this manuscript. All authors contributed to the article and approved the submitted version.

## Conflict of Interest

The authors declare that the research was conducted in the absence of any commercial or financial relationships that could be construed as a potential conflict of interest.
